# One-step loop-mediated isothermal amplification system for *Mycobacterium marinum* detection

**DOI:** 10.1128/spectrum.02906-24

**Published:** 2025-06-11

**Authors:** Kayo Okumura, Yuji Miyamoto, Satoshi Mitarai, Manabu Ato

**Affiliations:** 1 Research Center for Biosafety, Laboratory Animal and Pathogen Bank, National Institute of Infectious Diseases, Japan Institute for Health Security, Aoba-cho, Higashimurayama, Tokyo, Japan; 2 Leprosy Research Center, National Institute of Infectious Diseases, Japan Institute for Health Security, Aoba-cho, Higashimurayama, Tokyo, Japan; 3 Department of Mycobacterium Reference and Research, Research Institute of Tuberculosis, Japan Anti-tuberculosis Association 543860, Kiyose, Tokyo, Japan; Taichung Veterans General Hospital, Taichung, Taiwan, China

**Keywords:** *Mycobacterium marinum*, cutaneous NTM, one-step LAMP, hydroxy naphthol blue, specificity, sensitivity

## Abstract

*Mycobacterium marinum* is the etiological agent of cutaneous nontuberculous mycobacteriosis, inducing granuloma formation in human skin. Other mycobacterial species, including *Mycobacterium ulcerans*, *Mycobacterium chelonae*, and *Mycobacterium fortuitum*, also cause cutaneous infections, with *M. ulcerans* and *M. ulcerans* subsp. *shinshuense* showing close genetic relatedness to *M. marinum. M. ulcerans* is a recognized causative agent of Buruli ulcer, and numerous molecular detection and diagnostic methods have been established. However, there is a lack of specific detection methods for *M. marinum*. Currently, confirming *M. marinum* infection requires 16S rRNA or housekeeping gene sequencing, which is labor-intensive and time-consuming. In this study, we developed a one-step colorimetric loop-mediated isothermal amplification assay for simple and specific detection of *M. marinum*. The results were confirmed by visual inspection of color change in the reaction solution, eliminating the need for specialized equipment. Our proposed workflow can be completed within 60 minutes from sample preparation to detection with high sensitivity. To the best of our knowledge, the loop-mediated isothermal amplification assay developed in this study represents the only test capable of specifically detecting *M. marinum* utilizing only the loop-mediated isothermal amplification reaction. Consequently, this loop-mediated isothermal amplification assay has the potential for implementation in routine diagnosis of *M. marinum*.

**IMPORTANCE:**

*Mycobacterium marinum* and *Mycobacterium ulcerans*, which are frequently reported as etiological agents of cutaneous nontuberculous mycobacteria, exhibit similar characteristics in terms of symptomatology and patient demographics. However, there is currently no simple identification method to distinguish between these two species. The one-step loop-mediated isothermal amplification method developed in this study represents a rapid diagnostic approach that yields results in a short time frame and does not necessitate specialized precision instruments, as the results can be visually determined. Consequently, the loop-mediated isothermal amplification method established in this study is anticipated to contribute significantly to the advancement of diagnostic techniques for cutaneous nontuberculous mycobacteria.

## INTRODUCTION

Since the first identification of *Mycobacterium marinum* in dead saltwater fish by Aronson in 1926 (1), this pathogen has been isolated from various sources, including infected patient specimens, fish, other amphibians, and animal carcasses. *M. marinum* has also been found in environmental reservoirs; *M. marinum* strains infecting humans were isolated from swimming pools, wells, rivers, and fish tanks (2). When Aronson (1) initially isolated this organism, infections were primarily associated with public swimming pools, where many patients presented with common granulomatous skin lesions, leading to the term “swimming pool granuloma” (3, 4). However, with improved sanitation, the route of infection has shifted. Recent outbreaks of *M. marinum* have been reported in New York fish markets (5, 6), with sporadic outbreaks primarily linked to homes for ornamental fish, fishermen, and outdoor activities in ponds and lakes (2, 3). Given that these infections often result from contact with ornamental fish, *M. marinum* infections should be considered zoonotic (7).

*M. marinum* is the leading cause of extrarespiratory human infections among nontuberculous mycobacteria (NTM) (7) and is responsible for cutaneous mycobacterial infections (3). Other NTM such as *Mycobacterium chelonae, Mycobacterium fortuitum*, *Mycobacterium haemophilum*, *Mycobacterium ulcerans,* and *M. ulcerans* subsp. *shinshuense* are also known to cause cutaneous infections (3, 8). Diagnosing cutaneous NTM infections typically requires lesion tissue biopsies to check for acid-fast bacilli (AFB) and cultures of tissue samples or materials from draining lesions (3, 5, 8). However, identifying bacilli after AFB staining in skin biopsies and culturing mycobacteria can be challenging. Therefore, a multidisciplinary approach combining histopathology, bacterial culture, and molecular analysis is essential for accurate diagnosis. Molecular techniques, including PCR sequencing and matrix-assisted laser desorption/ionization-time-of-flight (MALDI-TOF) mass spectrometry, have been developed to facilitate the diagnosis of cutaneous NTM. Nevertheless, the current MALDI-TOF mass algorithm exhibits limitations in differentiating between *M. marinum* and *M. ulcerans*.

Mycolactone, a macrolide toxin produced by *M. ulcerans,* is known for its cytotoxic, analgesic, and immunosuppressive properties, making it a significant virulence factor for this species (9, 10). Some *M. marinum* clinical isolates have also been found to produce mycolactone (11, 12). Thin-layer chromatography detection of mycolactones has assisted in identifying NTM species. Therefore, developing a diagnostic method that utilizes mycolactone production as an index while specifically detecting *M. marinum* would be ideal. Currently, a definitive diagnosis of *M. marinum*, *M. ulcerans*, and *M. ulcerans* subsp. *shinshuense* requires sequence analysis of the 16S rRNA or housekeeping genes (13). However, a rapid diagnostic method for *M. marinum* has not yet been fully established.

In this study, we aimed to develop a loop-mediated isothermal amplification (LAMP) method for specifically detecting *M. marinum* using purified DNA and crude cell lysates from bacterial colonies. Based on a comparative genomic analysis of NTM, we identified candidate genes uniquely present in the *M. marinum* genome as targets for *M. marinum* detection and subsequently developed a corresponding protocol. Our one-step LAMP system can be completed in a single tube and determined by endpoint colorimetric readouts, enabling rapid and simple detection of *M. marinum*.

## RESULTS

### Design of LAMP primers

**Fig 1:**
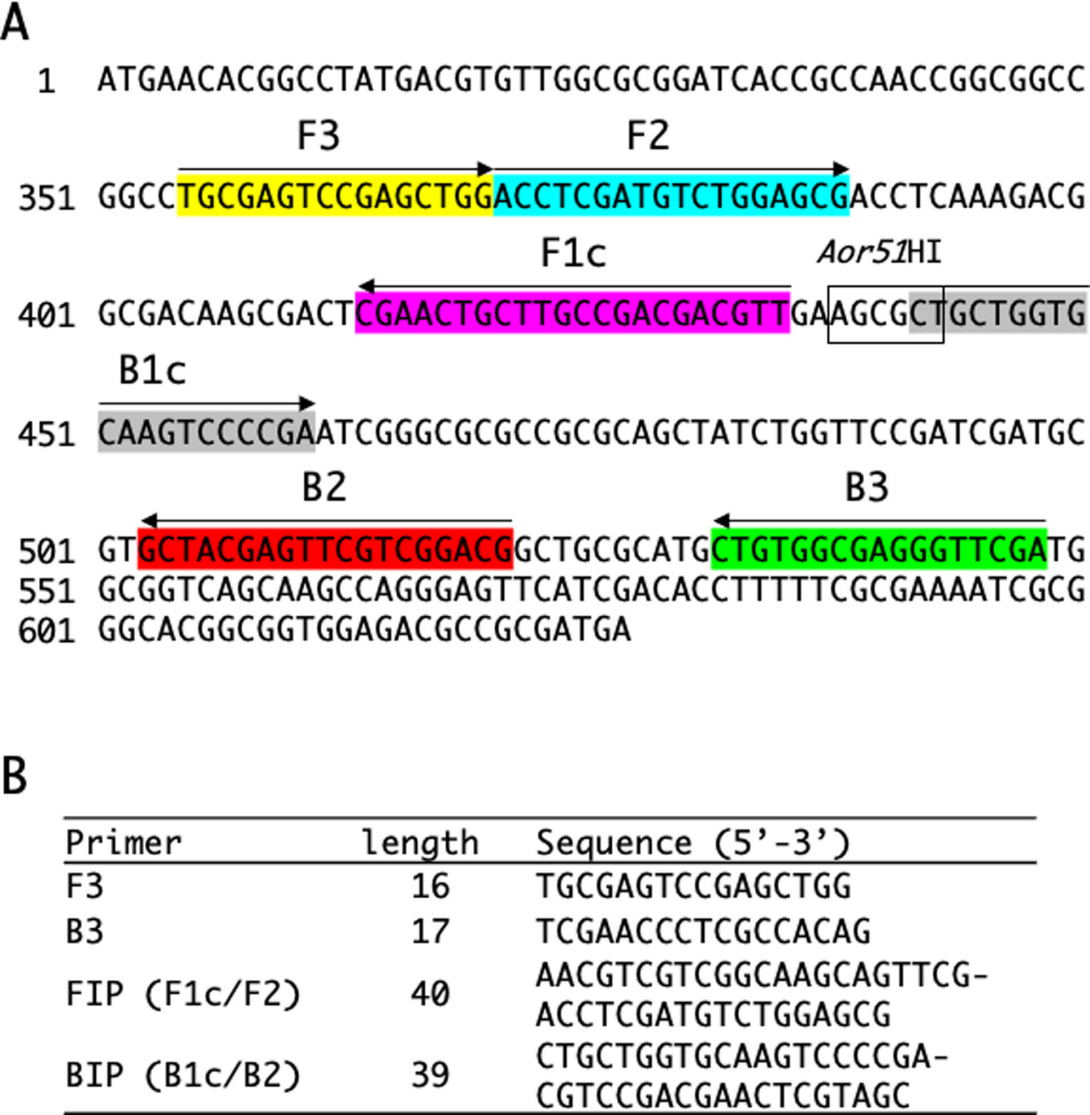
LAMP primer arrangements and sequences. (**A**) Numbers on the left indicate the position at the MMRN_19150 gene. The position of each primer sequence is indicated by highlighted areas and arrows. The restriction enzyme *Aor51*HI recognition sequence is enclosed in squares. (**B**) Sequences of the primers used and their lengths.

To construct a LAMP method for the specific detection of *M. marinum*, we compared genome sequences of *M. marinum* ATCC 927 and *M. ulcerans* ATCC 19423 to identify unique coding sequences (CDSs) specific to *M. marinum*. After selecting several candidate genes based on BLASTn analysis, we evaluated their similarity to genes found in species associated with cutaneous acid-fast bacillosis and pathogenic bacteria causing skin infections other than AFB. Based on this evaluation, we selected the MMRN_19150 gene, which encodes a hypothetical protein, as the target gene (Fig. 1). Owing to the genetic relatedness between *Mycobacterium pseudoshottsii*, an etiological agent of mycobacteriosis infections in fish, and *M. marinum*, the MMRN_19150 gene was identified in *M. pseudoshottsii*. Multiple candidate primer sets were generated using Primer Explorer V5 software, and after confirming the amplification of the target gene, a specific primer set for detecting *M. marinum* was finalized. Initially, five primer sets were selected as potential candidates, which were designed based on the genome sequence of *M. marinum* ATCC 927. However, the extraction of the target gene, the MMRN_19150 gene, for detection from various strains and subsequent alignment analyses revealed mismatched bases at primer annealing sites in certain strain sequences. To enhance the reliability of the target region amplification, BLAST analysis was conducted on the mycobacterial genomes to identify sequences similar to the primer sequences in other genomic regions. As a result of these analyses, corresponding primer sets were excluded. Subsequent to these screening procedures, we conducted LAMP reactions utilizing several remaining primer sets. Thus, preliminary tests were performed using genomic DNA from *M. marinum* ATCC 927, and the primers that successfully yielded amplified LAMP products without problems were selected as the final primer set. Details of the MMRN_19150 gene sequence and primer regions are shown in Fig. 1.

### Optimization of LAMP reactions

**Fig 2:**
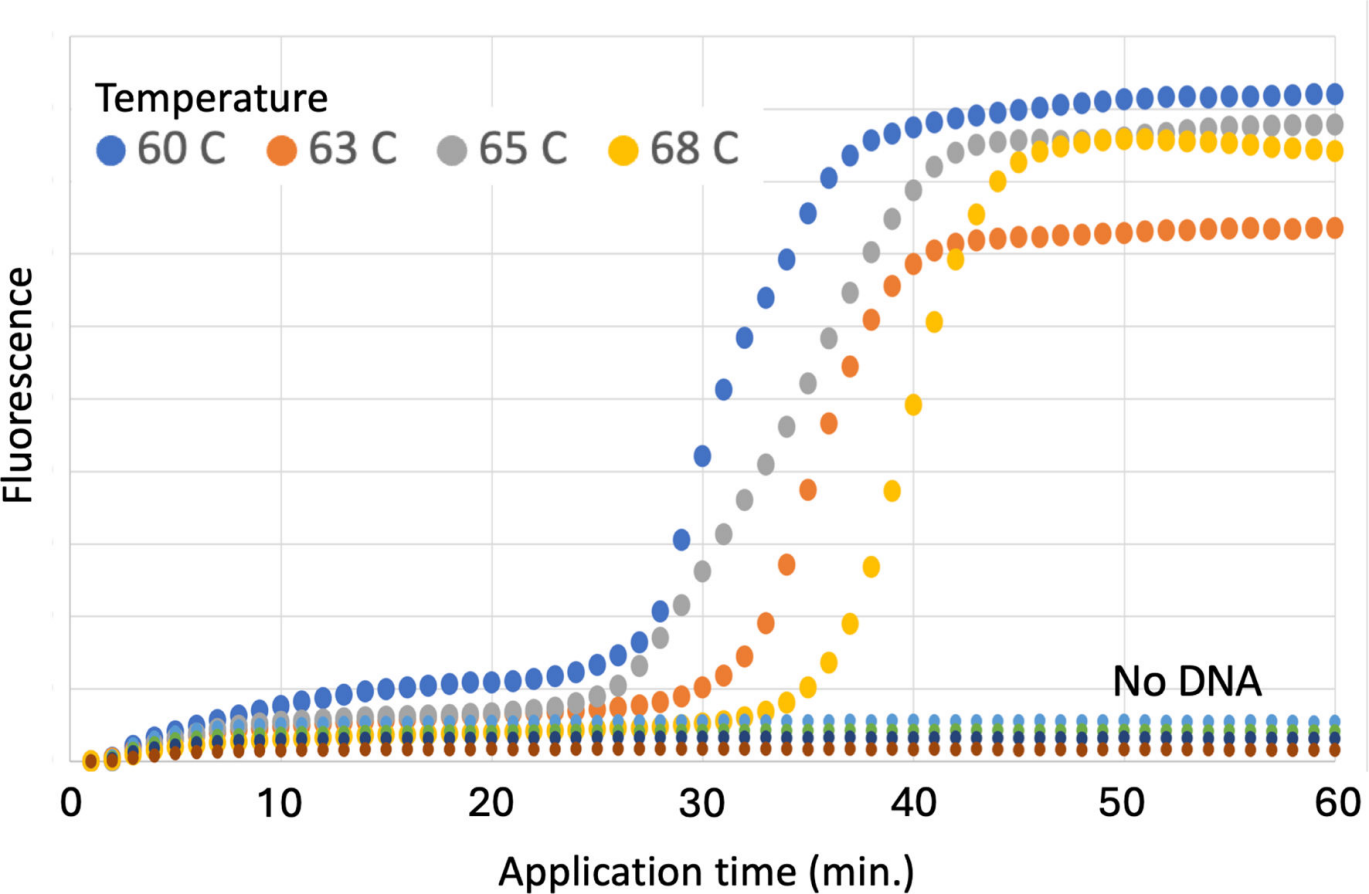
Comparison of fluorescence intensity at each reaction temperature. Plots of fluorescence intensities for amplification at reaction temperatures of 60°C, 63°C, 65°C, and 68°C. Horizontal axis represents reaction time (minutes). Vertical axis represents fluorescence intensity. Fluorescence intensity value at each temperature was measured without adding template DNA (no DNA).

**Fig 3:**
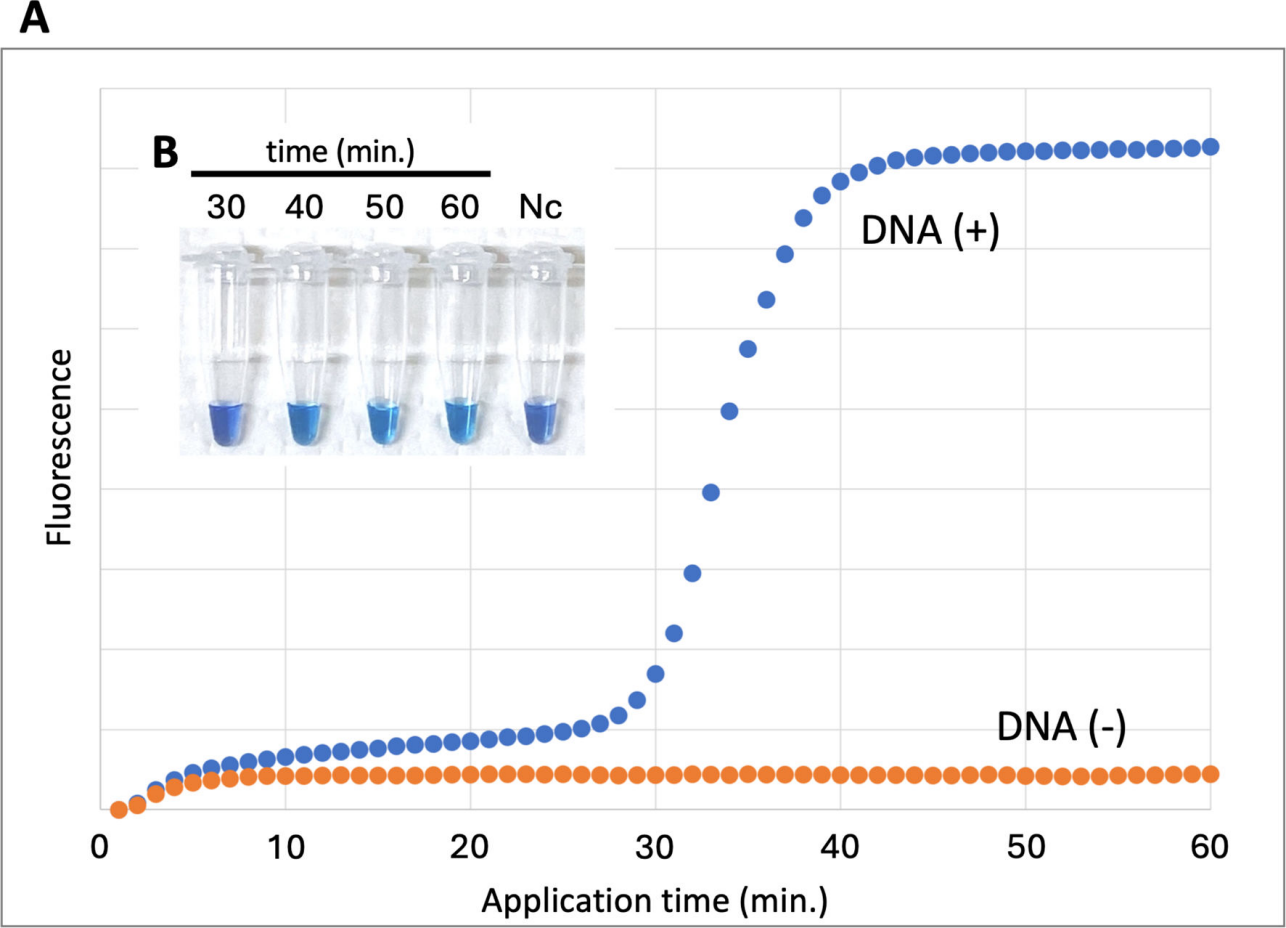
Comparison of fluorescence intensity at each reaction time. (**A**) Changes in fluorescence intensity over time are measured by real-time PCR at 60°C. “DNA (+)” and “DNA (−)” indicate conditions with or without the addition of template DNA. (**B**) HNB-LAMP reaction tubes at 30, 40, 50, and 60 minutes at 60°C; Nc represents the negative control sample (reaction for 60 minutes without the addition of template DNA).

To optimize the LAMP reaction conditions, we performed the reactions under multiple temperatures (60°C, 63°C, 65°C, and 68°C) while monitoring fluorescence intensity using real-time PCR for 60 minutes. The highest and earliest increase in fluorescence intensity was observed at 60°C (Fig. 2). At this temperature, fluorescence intensity began to rise approximately 30 minutes after the initiation of the reaction and reached maximum levels in about 40 minutes (Fig. 2 and 3A). Furthermore, the reactions were monitored at the same temperatures using 10-fold serial dilutions of template DNA, ranging from 5 ng to 50 pg. The findings indicated no variation in these trends, and an early amplification reaction was consistently observed at 60°C across all concentrations (Fig. S1). The reaction time was validated using a hydroxy naphthol blue (HNB) reagent, with results consistent with real-time PCR and fluorescent dye: a negative reaction at 30 minutes and a positive HNB reaction (blue color) after 40 minutes (Fig. 3B).

### Digestion of LAMP amplicons

**Fig 4:**
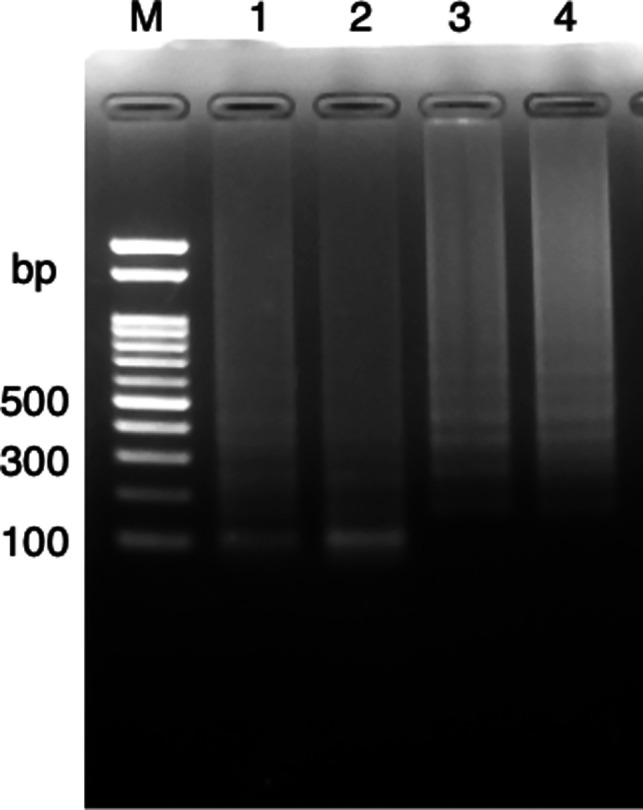
Analysis of LAMP amplification products by restriction enzyme digestion. Lanes are labeled as follows: Lane M: 100 bp ladder marker. Lane 1: *M. marinum* ATCC 927 DNA amplification product (*Aor51*HI digest). Lane 2: *M. pseudoshottsii* JCM 15466 DNA amplification product (*Aor51*HI digest). Lane 3: *M. marinum* ATCC 927 DNA amplification product (uncut). Lane 4: *M. pseudoshottsii* JCM 15466 DNA amplification product (uncut).

Post-LAMP reaction, the products were digested with the restriction enzyme Aor51HI, which has a recognition site within the target region. The restriction enzyme digestion changed the amplified signal of LAMP from the characteristic ladder band pattern to a more convergent pattern (Fig. 4), confirming that our primer set amplified the correct genomic region.

### LAMP specificity and sensitivity

**Fig 5:**
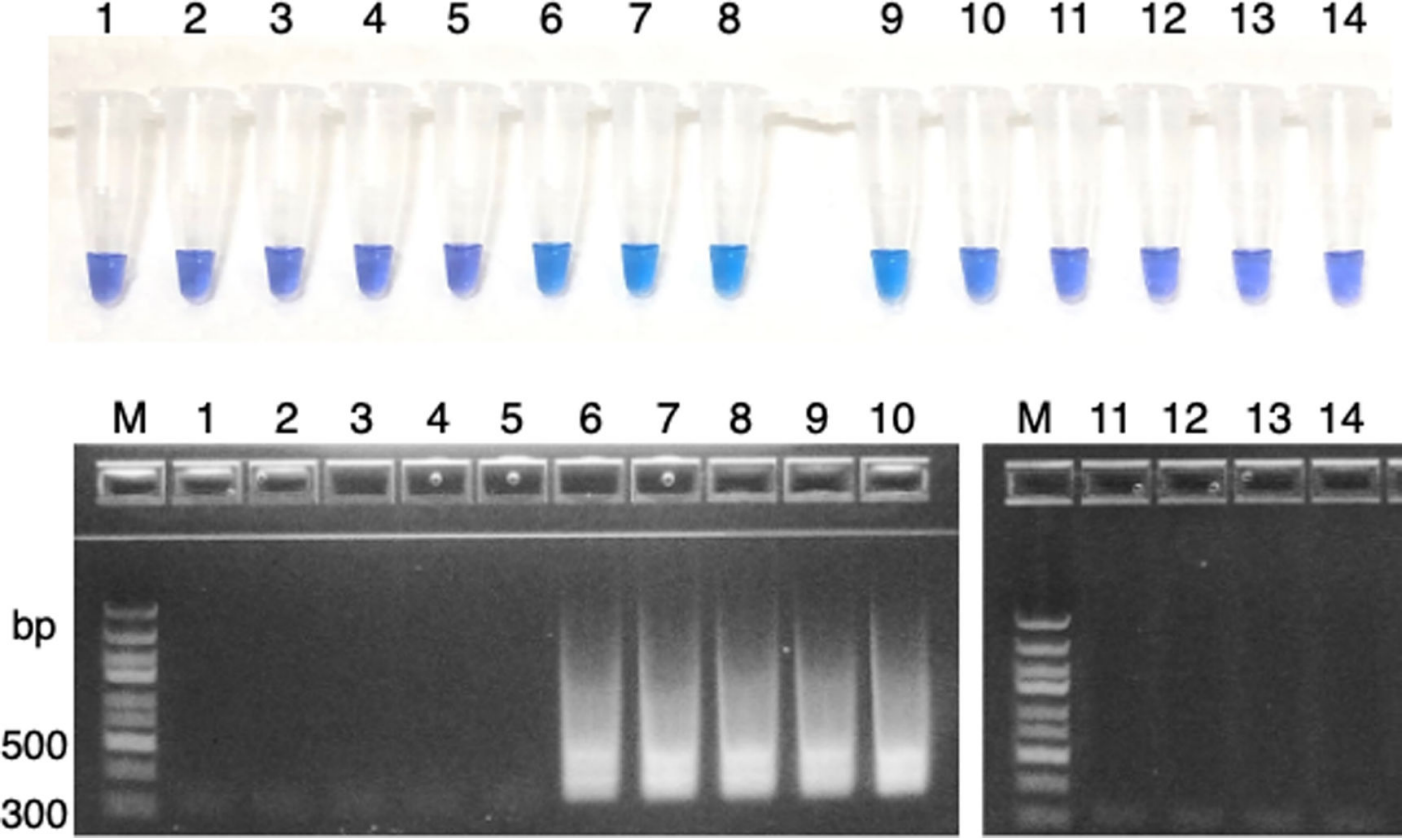
Specificity of LAMP assay for the detection of *M. marinum*. HNB-LAMP reaction solution indicates positive (blue) and negative (purple). Each amplified product was analyzed by agarose gel electrophoresis. Lanes (tubes) are labeled as follows: lane M: 100 bp ladder marker; lane 1: *Mycobacterium abscessus* JCM 13569; lane 2: *Mycobacterium avium* 104; lane 3: *M. chelonae* JCM 6388; lane 4: *M. fortuitum* clinical isolate; lane 5: *M. haemophilum* ATCC 29548; lane 6: *M. marinum* ATCC 927; lane 7: *M. marinum* clinical isolate LRC-1; lane 8: *M. marinum* clinical isolate LRC-9; lane 9: *M. marinum* clinical isolate LRC-16; lane 10: *M. pseudoshottsii* JCM 15466; lane M: DNA size marker (100 bp ladder); lane 11: *Mycobacterium smegmatis* mc^2^155; lane 12: *M. ulcerans* Agy99; lane 13: *M. ulcerans* subsp. *shinshuense* ATCC 33728; and lane 14: negative control (no DNA).

To assess the specificity of our method for detecting *M. marinum*, we performed the same experiments using skin isolates of AFB and other bacteria known to cause skin infections (Table 1). Amplification was observed for *M. marinum* and for the etiological agent *M. pseudoshottsii*, which is present in fish; notably, no amplification was detected for other bacterial DNA (Fig. 5). When using DNA from *M. marinum* and *M. pseudoshottsii* as the template, the reaction solution turned blue, indicating a positive result. In contrast, DNA from other bacteria, including *M. ulcerans* and *M. ulcerans* subsp. *shinshuense*, caused the reaction solution to turn purple, indicating a negative result. These outcomes were corroborated by precast agarose gel electrophoresis, where a characteristic ladder-like pattern was observed with DNA from *M. marinum* and *M. pseudoshottsii* but not with other DNA (Fig. 5). Notably, *M. ulcerans* was not detected using this LAMP method, whereas *M. marinum* was consistently detected. To validate the robustness of our LAMP method, we increased the number of *M. ulcerans* strains to match that of *M. marinum* strains and conducted the analysis. Moreover, to comprehensively verify the specificity of the LAMP, in addition to examining *M. ulcerans* subsp. *shinshuense*, which is endemic to Japan, we examined a diverse range of *M. ulcerans* isolates from various geographical origins outside Japan, including Africa, Oceania, and Asia.

**TABLE 1:** Specificity of HNB-LAMP on *M. marinum* strains, species of *Mycobacterium,* and other bacteria^*a*^

Species	Source (strain)	No. of strains	No. of positive strains
*Mycobacterium* strains			
*Mycobacterium abscessus*	JCM 13569	1	0
*Mycobacterium avium*	104	1	0
*M. chelonae*	JCM 6388	1	0
*M. fortuitum*	Clinical isolates	1	0
*M. haemophilum*	ATCC 29548	1	0
*M. marinum*	ATCC 927	1	1
	Clinical isolates	43	43
*M. pseudoshottsii*	JCM 15466	1	1
*M. smegmatis*	mc^2^155	1	0
*M. ulcerans*	Agy99	1	0
	Clinical isolates	10	0
*M. ulcerans* subsp. *shinshuense*	ATCC 33728	1	0
	Clinical isolates	24	0
Others			
*Pseudomonas aeruginosa*	JCM 5962	1	0
*Staphylococcus aureus*	N315	1	0
*Staphylococcus capitis*	JCM 2420	1	0
*Staphylococcus cohnii*	JCM 2417	1	0
*Staphylococcus epidermidis*	JCM 2414	1	0
*Staphylococcus haemolyticus*	JCM 2416	1	0
*Staphylococcus hominis*	JCM 31912	1	0
*Staphylococcus saccharolyticus*	JCM 1768	1	0
*Staphylococcus saprophyticus*	JCM 2427	1	0
*Staphylococcus warneri*	JCM 2415	1	0
*Streptococcus agalactiae*	JCM 5671	1	0
*Streptococcus pyogenes*	JCM 5674	1	0

^*a*^ JCM, Japan Collection of Microorganisms, RIKEN BioResource Center, Ibaraki, Japan. ATCC, American Type Culture Collection, Manassas, VA, USA. Clinical isolates are strains isolated in Japan and originated from the following three facilities: National Institute of Infectious Diseases, Tokyo, Japan; the Research Institute of Tuberculosis, Tokyo, Japan; and SRL, Tokyo, Japan.

**Fig 6:**
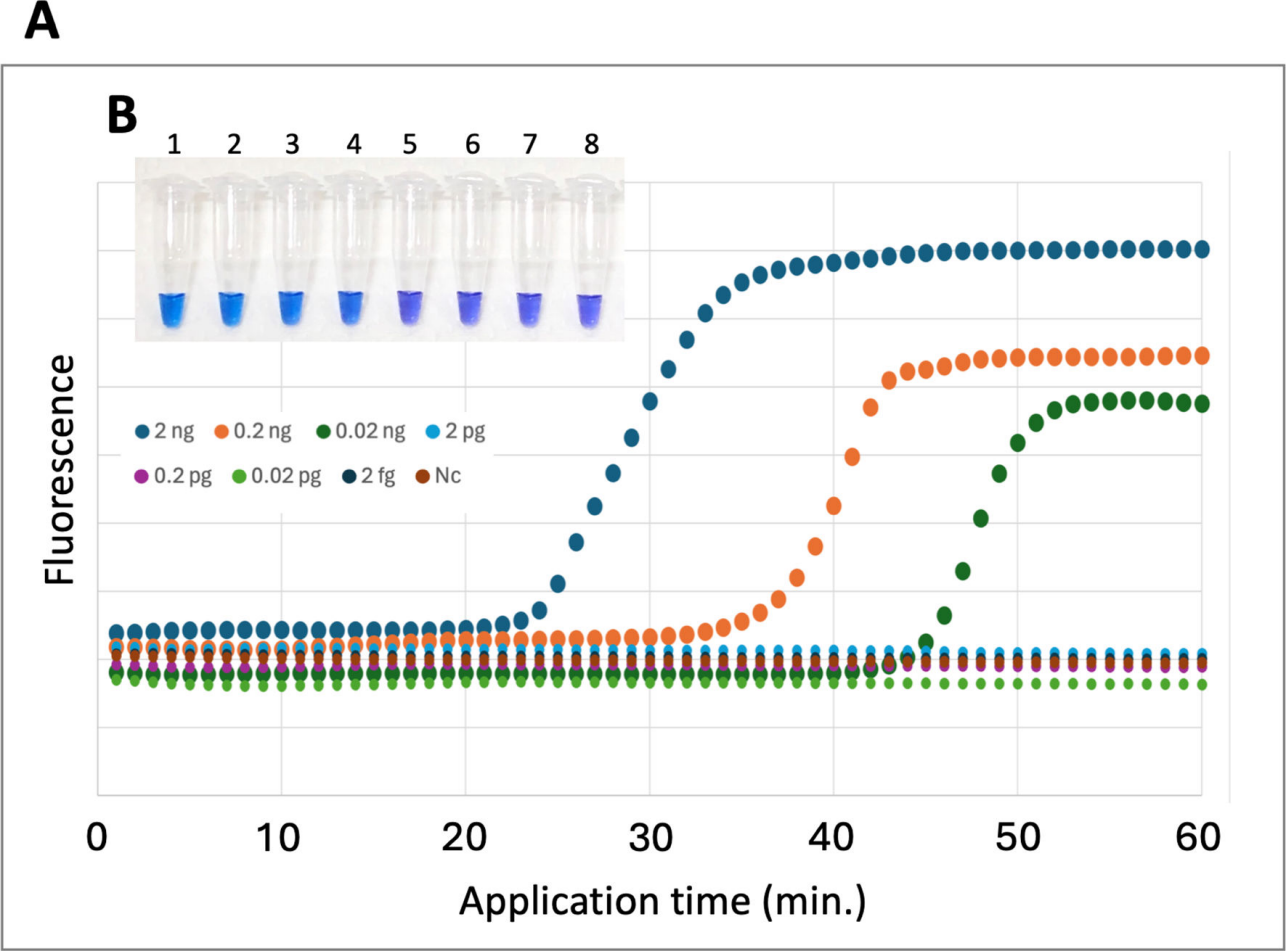
Sensitivity of LAMP assay for the detection of *M. marinum*. Evaluation of LAMP assay’s response to 10-fold serial dilutions of DNA, ranging from 2 ng/µL to 2 fg/μL, using real-time PCR (**A**) and HNB-LAMP (**B**). (**A**) Fluorescence intensity for each DNA concentration monitored over 60 minutes. Horizontal axis represents reaction time (minutes). Vertical axis represents fluorescence intensity. (**B**) Differences in reaction at each DNA concentration in HNB-LAMP. Tube 1: 2 ng/µL DNA; tube 2: 0.2 ng/µL DNA; tube 3: 0.02 ng/µL DNA; tube 4: 2 pg/µL DNA; tube 5: 0.2 pg/µL DNA; tube 6: 0.02 pg/µL DNA; tube 7: 2 fg/μL DNA; and tube 8: negative control (no DNA).

We measured fluorescence intensity using real-time PCR and HNB-LAMP to evaluate detection sensitivity. Chromosomal DNA concentrations ranging from 2 ng/µL to 2 fg/μL of *M. marinum* were analyzed. The detection limit was 20 pg/µL for real-time PCR (Fig. 6A), and that of HNB-LAMP was 2 pg/µL (Fig. 6B), demonstrating that HNB-LAMP had higher detection sensitivity compared with real-time PCR.

## DISCUSSION

The LAMP method developed in this study enabled us to distinguish *M. marinum* from other causative organisms of cutaneous NTM infection. Due to the simplicity of the reaction, which involves two steps at temperatures of 60°C and 95°C, the assay can be performed using only a water bath incubator or heating blocks. Furthermore, the method uses a LAMP reaction solution and a colorimetric indicator, HNB, allowing results to be visually judged without requiring specialized equipment such as a UV transilluminator. As the LAMP reaction proceeds, magnesium pyrophosphate is produced as a byproduct, decreasing Mg^2+^ ions in the reaction solution. HNB, a metal indicator, monitors this decrease, causing the color of the LAMP reaction solution to change from purple (negative) to sky blue (positive) (14). Because this method is simple and does not require sophisticated instruments, it can be widely implemented in various settings, including environments with inadequate equipment. Notably, when cutaneous NTM were subjected to the one-step LAMP assay, no cross-amplification was observed except for *M. pseudoshottsii*. Given that *M. pseudoshottsii* is an etiological agent of mycobacteriosis in fish, considering NTM that infects humans, this LAMP method demonstrates specificity for *M. marinum* detection, enabling diagnosis without the need for specialized reactions such as post-LAMP procedures, including restriction enzyme digestion. Importantly, this method does not require PCR reaction reagents and other associated reagents that are required for conventional 16S rRNA sequencing analysis. Moreover, it does not entail the time and labor associated with subsequent PCR analyses. Furthermore, the absence of expensive equipment such as sequencers eliminates the need to consider operational costs related to the maintenance and management of analytical instruments. The results of this analysis were consistent with the expected outcomes; specifically, LAMP-positive results were obtained from all *M. marinum* and *M. pseudoshottsii* isolates tested, while LAMP-negative results were obtained from all other strains/isolates, including pathogenic bacteria other than acid-fast bacilli, as well as *M. ulcerans*. We conducted LAMP experiments in three distinct laboratories, each utilizing a thermal cycler from a different manufacturer and a heat block incubator, and the results were consistent. However, it is important to note that our experimental conditions were rigorously controlled in terms of temperature and other variables, and the analyses were performed by a limited number of researchers. Therefore, future studies are warranted to verify whether similarly stable results can be obtained using a larger number of clinical isolates across diverse experimental settings, ranging from laboratories with less sophisticated experimental environments to facilities maintaining comparable standards to those of our facility. The actual sensitivity and specificity can be assessed once a substantial amount of test data has been accumulated through blinded studies and large-scale investigations, such as those conducted across various countries, research institutions, and laboratories.

The sensitivity of the LAMP method constructed in this study was 2 pg/µL, comparable to (0.5 pg/µL) or higher (48 pg/µL) than that of other LAMP methods for detecting AFB (15, 16). Although previous reports indicate that *M. marinum* may not always be detected through culture-based diagnosis from biopsy (5, 17), bacterial culture remains the gold standard for diagnosing NTM infection (5). Consequently, when colonies of approximately one loop are obtained following the isolation of clinical samples, the detection sensitivity at this level is sufficient to identify *M. marinum*. Given that *M. marinum* grows faster than other slow-growing mycobacteria, such as *M. ulcerans* and *M. avium*, colonies can typically be obtained within 2 weeks and, in some cases, within 1 week. Once a colony is obtained, our LAMP method can help determine with high accuracy whether the clinical samples are *M. marinum-*positive.

The optimal antibiotic therapy for *M. marinum* infections has not yet been established; therefore, treatments often involve empiric antibiotics and conventional therapies. Monotherapy with clarithromycin, trimethoprim, or ciprofloxacin is considered adequate for superficial cutaneous infections, while combination therapy with two drugs may be more effective for deeper infections (2). Additionally, *M. marinum* is naturally multidrug-resistant (18), often showing resistance to anti-tuberculosis drugs such as isoniazid, ethambutol, and pyrazinamide (3). In contrast, a treatment strategy has been established for *M. ulcerans*, the causative agent of Buruli ulcer (BU), which is genetically closely related to *M. marinum*. The World Health Organization recommends combinations of clarithromycin and rifampicin or streptomycin and rifampicin for treating BU (19). Thus, although these two bacterial species are genetically related and can cause cutaneous NTM infections, their treatment strategies differ. Little is known about the risk factors and clinical and microbiological characteristics of each pathogen in cutaneous NTM infections in Japan (20). Therefore, accurate differentiation between these species is crucial for administering appropriate antibiotic therapy and shortening the duration of treatment. Moreover, BU is a neglected tropical disease, and globally, the third most prevalent mycobacterial disease, following tuberculosis and leprosy (19). BU presents significant treatment challenges when its diagnosis is delayed, resulting in ulcer formation and potentially permanent disability. Therefore, early diagnosis followed by appropriate antibiotic administration is crucial for minimizing the morbidity associated with BU. In Japan, *M. marinum* infections exhibit the highest prevalence, with a limited number of BU cases reported annually (20). Hence, establishing an accurate diagnostic modality for *M. marinum* could facilitate early diagnosis of BU, which is crucial for effective BU management. Furthermore, this phenomenon is not confined to infections caused by *M. marinum*; in skin infections, secondary infections by organisms other than the primary causative agents are also possible. The LAMP method did not detect non-mycobacterial pathogens such as *Pseudomonas aeruginosa* and *Staphylococcus aureus* (Table 1), which are frequently isolated from the skin. This suggests that the LAMP method can effectively exclude the detection of secondary infectious organisms, thereby reducing the risk of misdiagnosis due to erroneous results.

IS*2404*-targeted PCR and qPCR are widely used as sensitive and specific molecular tests and are currently considered the gold standard for the definitive diagnosis of cutaneous NTM infections (21). Although IS*2404* has been used as a genetic marker for *M. ulcerans*, fish-derived *M. marinum* isolates have also been found to harbor IS*2404* (12). To date, no human-derived *M. marinum* harboring IS*2404* has been found; however, using the presence of IS*2404* as a criterion for identifying *M. ulcerans* or *M. marinum* poses some risk. Furthermore, *M. ulcerans* produces a unique macrolide called mycolactone, which has cytotoxic and immunosuppressive properties (10). Six protein-coding genes involved in mycolactone synthesis are located on a 174 kb giant plasmid (10). Of these genes, *mlsA1* (51 kb) and *mlsA2* (7 kb) encode mycolactone core-producing structures, and *mlsB* (42 kb) encodes a side chain enzyme (10). Studies have shown that *M. ulcerans* acquired this plasmid through horizontal gene transfer, and its deletion leads to a loss of pathogenicity (9, 10, 22). Some *M. marinum* strains also produce mycolactone, although their molecular structures differ slightly from those of *M. ulcerans* (12, 23). Thus, most diagnostic methods for cutaneous NTM have focused on detecting mycolactone, mycolactone-related genes, or IS*2404*. Given the shared gene possession between *M. marinum* and *M. ulcerans* and the heterogeneity of gene conservation among *M. marinum* strains, it is imperative to establish a detection method independent of IS*2404* and mycolactone-related genes.

To address these challenges, Tsai et al. (11) developed a detection method targeting the *mrsA* gene, independent of IS*2404* and mycolactone-related genes and located on the chromosomal genome. Their LAMP assay targeting the *mrsA* gene demonstrated favorable sensitivity and specificity, with a reaction time of less than 60 minutes. However, their target gene was conserved in *M. marinum* and *M. ulcerans* genomes (11). Consequently, the amplified product was subjected to restriction enzyme treatment, and differences in band patterns were utilized to determine the species of these two organisms. The method involves treating the amplified LAMP products with restriction enzymes and confirming the digested sample by electrophoresis. However, these procedures increase the risk of contamination and require laborious steps, such as tube opening, reagent addition, and confirmation by electrophoresis. Moreover, point mutations in the enzyme recognition sequence may alter cleavage patterns, potentially leading to unexpected outcomes.

In summary, the LAMP assay developed in this study was found to be specific for *M. marinum*. The results were obtained seamlessly, suggesting that this method is straightforward, reliable, and reproducible. To our knowledge, this is the first LAMP assay specifically designed to detect *M. marinum* using only the LAMP reaction. Therefore, our findings suggest that this LAMP assay has the potential for integration into routine diagnostic protocols for *M. marinum*.

## MATERIALS AND METHODS

### Strains and media

The bacterial strains of *Mycobacterium* spp. and other bacterial species used in this study are listed in Table 1. These strains were stored as glycerol stocks and cultured in appropriate media when used for analysis. Culture conditions, including incubation temperature, durations, and media used, are detailed in Table S1. All strains were verified for species identity through 16S rRNA gene sequencing, following the methods described by Nakanaga et al. (13) for *Mycobacterium* spp. and general experimental protocols for other bacteria.

### Preparation of template DNA

One loopful of bacteria from the medium was suspended in 100 µL of distilled water and heated at 95°C for 10 minutes. The suspensions were centrifuged at 8,000 × *g* for 5 minutes, and the supernatant was used as the DNA solution. These DNA solutions were stored at −30°C until further use. *M. marinum* ATCC 927 DNA was also extracted using a commercial kit to determine optimal LAMP reaction conditions. One loopful of bacteria was suspended in 100 µL of 7H9 medium and harvested by centrifugation at 8,000 × *g* for 5 minutes. The bacterial pellets were used to extract chromosomal DNA with the High Pure PCR Template Preparation Kit (Roche, Mannheim, Germany) according to the manufacturer’s instructions. DNA concentration and purity were assessed using NanoDrop One (ThermoFisher Scientific, Tokyo, Japan) and stored at −30°C until further use.

### LAMP primer design

To establish the primer settings, we used genomic sequence data from *M. marinum* ATCC 927 (GenBank accession number, AP018496.1) and *M. ulcerans* ATCC 19423 (GenBank accession number, NZ_CP092429.2). Target regions specific to *M. marinum* and the genome sequences of *M. marinum* and *M. ulcerans* were compared using Mauve software (version 2.4.0 https://darlinglab.org/mauve/mauve.html). Additionally, we confirmed the specificity of the target gene using the Nucleotide Basic Local Alignment Search Tool analysis using DNA from humans and other organisms. Once the target gene was identified, primers were designed using Primer Explorer V5 software (https://primerexplorer.eiken.co.jp/) according to the manufacturer’s instructions, based on the nucleotide sequence of the hypothetical protein gene (locus_tag MMRN_19150) of *M. marinum* strain ATCC 927.

### Optimization of LAMP reactions

Reactions were performed at various temperatures to optimize LAMP reaction conditions, and fluorescence intensity was monitored using a QuantStudio 7 Pro Real-Time PCR System (ThermoFisher Scientific). The reactions were performed using 2× LAMP Master Mix (Eiken Chemical, Tokyo, Japan). The reaction mixture was prepared by adding 12.5 µL of 2× Reaction Master Mix (Eiken Chemical), 40 pmol of FIP and BIP primers, 5 pmol of F3 and B3 primers, 50 ng of template DNA (from *M. marinum* ATCC 927 purified with the commercial kit), and sterile water to a final volume of 25 µL. Amplification was conducted and monitored at different temperatures (60°C, 63°C, 65°C, and 68°C) for 60 minutes. After amplification, the enzyme was inactivated by heating at 95°C for 2 minutes. Furthermore, serial dilutions (10-fold) of the template DNA were observed, ranging from 5 ng to 50 pg.

### Specificity of LAMP reactions

To assess the specificity of the constructed LAMP system to detect *M. marinum*, we performed the same experiment using a Loopamp DNA Amplification Kit (Eiken Chemical) with cell lysates from clinical isolates of *M. marinum*, other *Mycobacterium* spp., and pathogenic bacteria causing skin infections (Table 1). The reaction mixture was prepared by adding 12.5 µL of 2× Reaction Mix (40 mM Tris-HCl [pH 8.8], 20 mM KCl, 16 mM MgSO_4_, 20 mM (NH_4_)_2_SO_4_, 0.2% Tween20, 1.6 M Betaine, and 2.8 mM/each dNTP), 40 pmol of FIP and BIP primers, 5 pmol of F3 and B3 primers, 1 µL of *Bst* DNA Polymerase, 120 µM HNB (hydroxy naphthol blue) (Dojindo Laboratories, Tokyo, Japan), 50 ng of template DNA (for *M. marinum* ATCC 927) or 2 µL of 10-fold-diluted cell lysates (for bacteria other than *M. marinum* ATCC 927), and sterile water to make a total volume of 25 µL. Reactions were performed using a thermal cycler with amplification at 60°C for 60 minutes, followed by enzyme inactivation at 95°C for 2 minutes. The LAMP results were visually assessed based on the color changes (from purple to blue) of the HNB indicator.

### Digestion of LAMP amplicons

To verify target fragment amplification, LAMP amplification products (amplicons) were digested using the restriction enzyme *Aor*51HI, which has a restriction site in the target sequence (Fig. 1). The reaction mixture, with a total volume of 10 µL, included 1 µL *Aor*51HI (10 U/µL, Takara Bio Inc., Shiga, Japan), 10× M Buffer (1 µL), LAMP amplicon (1 µL), and sterile water (7 µL). The mixture was incubated at 37°C overnight and then electrophoresed using a 2% TAE agarose gel to visualize band patterns.

### Sensitivity of LAMP reactions

The sensitivity of the LAMP system was evaluated by preparing 10-fold serial dilutions of DNA from *M. marinum* ATCC 927. The LAMP reactions were performed using the Loopamp DNA Amplification Kit (Eiken Chemical) under previously described conditions. After the amplification, the products were confirmed by observing the color change of HNB. The detection limit was further assessed using 2× LAMP Master Mix and a Real-Time PCR System, maintaining the previously described conditions.

### Analysis of amplification products

In addition to the colorimetric changes with HNB, LAMP reaction products were analyzed by agarose gel electrophoresis using a precast system (E-Gel Power Snap Electrophoresis System, ThermoFisher Scientific). Electrophoresis was performed according to the manufacturer’s instructions with some modifications. Briefly, 2 µL of each amplified product was mixed with 8 µL of E-Gel Sample Loading Buffer (Invitrogen, Tokyo, Japan) and loaded into each well of E-Gel EX 1% agarose gel or E-Gel EX 1% double comb agarose gel to confirm the LAMP products. After 10–12 minutes of electrophoresis, the presence or absence of a signal was evaluated.

## Data Availability

All data generated or analyzed during this study are included in this published article.

## SUPPLEMENTAL MATERIAL

Supplemental material spectrum.02906-24-s0001.pdf Table S1; Fig. S1.

ASM does not own the copyrights to Supplemental Material that may be linked to, or accessed through, an article. The authors have granted ASM a non-exclusive, world-wide license to publish the Supplemental Material files. Please contact the corresponding author directly for reuse.
