# Supplementary material for: One-step loop-mediated isothermal amplification system for Mycobacterium marinum detection
Source: Microbiol Spectr. 2025 Jun 11;13(7):e02906-24. doi: 10.1128/spectrum.02906-24 (PMC12211018; doi:10.1128/spectrum.02906-24)
Supplement: Supplemental material — Table S1; Fig. S1. [file spectrum.02906-24-s0001.pdf]

Table S1 Culture conditions of *Mycobacterium* spp. and other bacteria.

Species	Source (strain)	Medium used	Temperature	Incubation periods
Mycobacterium strains				
M. abscessus	JCM 13569	Ogawa medium	37 °C	18 hours
M. avium	104	Ogawa medium	37 °C	21 days
M. chelonae	JCM 6388	Ogawa medium	37 °C	7 days
M. fortuitum	Clinical isolates	Ogawa medium	37 °C	7 days
M. haemophilum	ATCC 29548	Middlebrook 7H9 broth with 10% OADC + hemin	32 °C	4 weeks
M. marinum	ATCC 927	Ogawa medium	32 °C	10 days
	Clinical isolates	Ogawa medium	32 °C	10 days
M. pseudoshottsii	JCM 15466	Ogawa medium	32 °C	4 weeks
M. smegmatis	mc^2155	Ogawa medium	37 °C	3 days
M. ulcerans	Agy99	Ogawa medium	32 °C	8 weeks
	Clinical isolates	Ogawa medium	32 °C	8 weeks
M. ulcerans subsp. *shinshuense*	ATCC 33728	Ogawa medium	32 °C	8 weeks
	Clinical isolates	Ogawa medium	32 °C	8 weeks
Others				
Pseudomonas aeruginosa	JCM 5962	BHI plate	37 °C	18 hours
Staphylococcus aureus	N315	BHI plate	37 °C	18 hours
Sta. capitis	JCM 2420	BHI plate	37 °C	18 hours
Sta. cohnii	JCM 2417	BHI plate	37 °C	18 hours
Sta. epidermidis	JCM 2414	BHI plate	37 °C	18 hours
Sta. haemolyticus	JCM 2416	BHI plate	37 °C	18 hours
Sta. hominis	JCM 31912	BHI plate	37 °C	18 hours
Sta. saccharolyticus	JCM 1768	BHI broth	37 °C (anaerobic)	18 hours
Sta. saprophyticus	JCM 2427	BHI plate	37 °C	18 hours
Sta. warneri	JCM 2415	BHI plate	37 °C	18 hours
Streptococcus agalactiae	JCM 5671	BHI plate	37 °C	18 hours
Str. pyogenes	JCM 5674	BHI plate	37 °C	18 hours

BHI, Brain-Heart Infusion

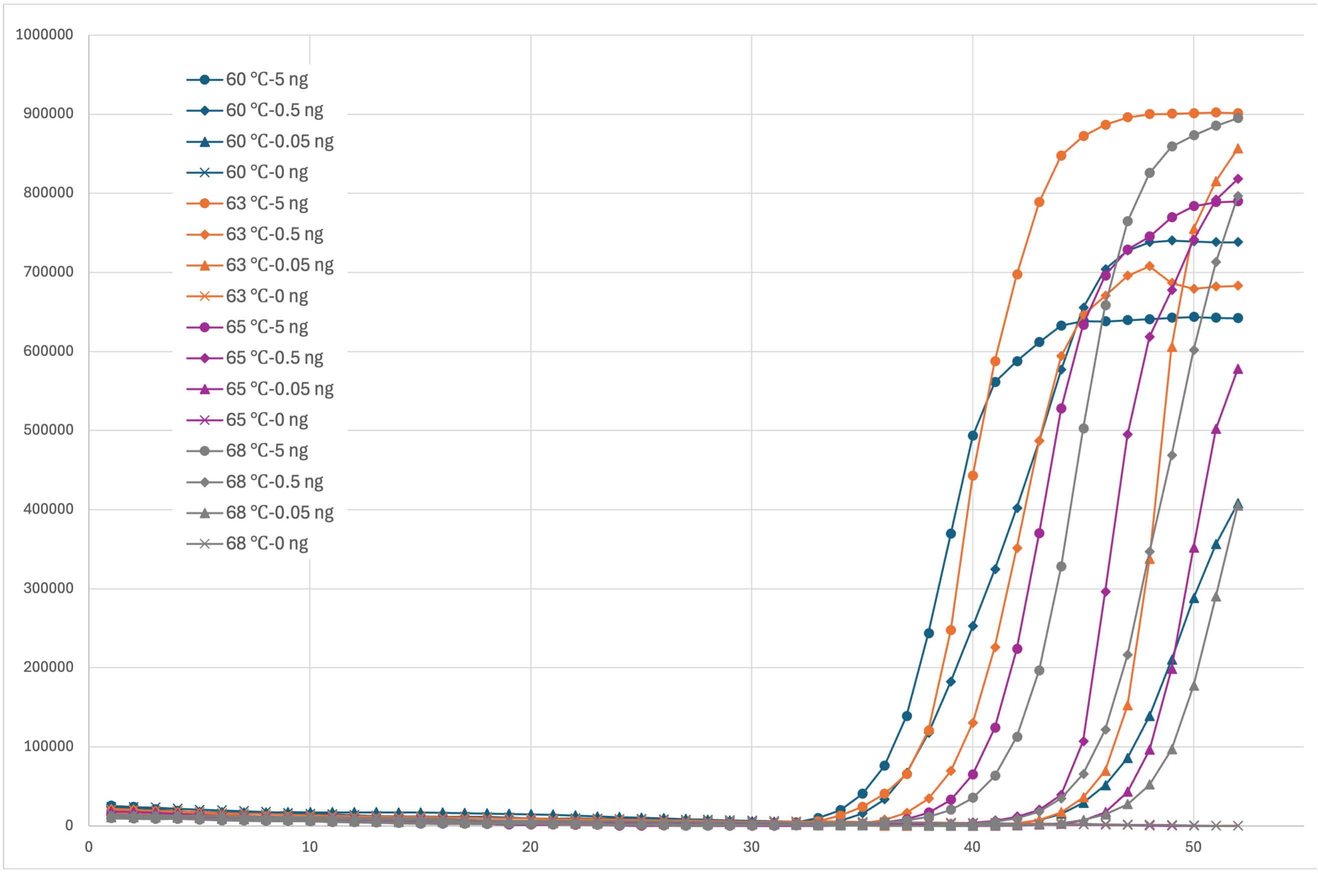

1000000
900000
800000
700000
600000
500000
400000
300000
200000
100000
0
0
10
20
30
40
50
60 °C-5 ng
60 °C-0.5 ng
60 °C-0.05 ng
60 °C-0 ng
63 °C-5 ng
63 °C-0.5 ng
63 °C-0.05 ng
63 °C-0 ng
65 °C-5 ng
65 °C-0.5 ng
65 °C-0.05 ng
65 °C-0 ng
68 °C-5 ng
68 °C-0.5 ng
68 °C-0.05 ng
68 °C-0 ng

Fig. S1

Fig. S1 | Comparison of fluorescence intensity at each reaction temperature.

Plots of fluorescence intensities for amplification at reaction temperatures of 60, 63, 65, and 68 °C. Evaluation of LAMP assay's response to 10-fold serial dilutions of DNA, ranging from 0.2 ng/μL to 2 pg/μL, using real-time PCR. The fluorescence intensity value at each temperature was also measured without adding template DNA (No DNA). The horizontal axis represents reaction time (min). The vertical axis represents fluorescence intensity.
